# Cardiac Rehabilitation Models around the Globe

**DOI:** 10.3390/jcm7090260

**Published:** 2018-09-07

**Authors:** Gabriela Lima de Melo Ghisi, Ella Pesah, Karam Turk-Adawi, Marta Supervia, Francisco Lopez Jimenez, Sherry L. Grace

**Affiliations:** 1 Cardiovascular Prevention and Rehabilitation Program, Toronto Rehabilitation Institute, University Health Network, University of Toronto, Toronto, ON M5G2A2, Canada; gabriela.meloghisi@uhn.ca; 2 School of Kinesiology and Health Science, York University, Toronto, ON M3J1P3, Canada; ellap@my.yorku.ca; 3 Public Health Department, College of Health Sciences, Qatar University, Al Jamiaa St, Doha, P.O. Box 2713, Qatar; kadawi@brandeis.edu; 4 Gregorio Marañón General University Hospital, Gregorio Marañón Health Research Institute, 28007 Madrid, Spain; msuperviapola@gmail.com; 5 Mayo Clinic, Rochester, MN 55905, USA; lopez@mayo.edu

**Keywords:** cardiac rehabilitation, surveys and questionnaires, international health, patient education as topic

## Abstract

Alternative models of cardiac rehabilitation (CR) delivery, such as home or community-based programs, have been developed to overcome underutilization. However, their availability and characteristics have never been assessed globally. In this cross-sectional study, a piloted survey was administered online to CR programs globally. CR was available in 111/203 (54.7%) countries globally; data were collected in 93 (83.8% country response rate). 1082 surveys (32.1% program response rate) were initiated. Globally, 85 (76.6%) countries with CR offered supervised programs, and 51 (45.9%; or 25.1% of all countries) offered some alternative model. Thirty-eight (34.2%) countries with CR offered home-based programs, with 106 (63.9%) programs offering some form of electronic CR (eCR). Twenty-five (22.5%) countries with CR offered community-based programs. Where available, programs served a mean of 21.4% ± 22.8% of their patients in home-based programs. The median dose for home-based CR was 3 sessions (Q25−Q75 = 1.0–4.0) and for community-based programs was 20 (Q25–Q75 = 9.6–36.0). Seventy-eight (47.0%) respondents did not perceive they had sufficient capacity to meet demand in their home-based program, for reasons including funding and insufficient staff. Where alternative CR models are offered, capacity is insufficient half the time. Home-based CR dose is insufficient to achieve health benefits. Allocation to program model should be evidence-based.

## 1. Introduction

Cardiac rehabilitation (CR) is an outpatient model of secondary preventive care primarily delivered in clinical settings. Given participation is associated with a 26% reduction in cardiovascular mortality and 18% reductions in re-hospitalization compared with controls [1]; it is a Class I Level A recommendation in clinical practice guidelines [2,3]. However, CR remains grossly under-utilized, particularly when juxtaposed against other guideline recommendations for cardiovascular disease (CVD) patients [4]. The reasons include patient-related factors such as geographic access, cost (including for transportation to sessions), and time conflicts due to return-to-work and family obligations [5,6], as well as health system-related factors such as insufficient capacity (i.e., 18,936,405 more CR “spots” needed to treat incident ischemic heart patients globally annually) [7]. To mitigate these barriers, alternative models have been developed, where CR is delivered in non-clinical settings, such as the home or community.

While there is no universally-agreed upon definition of home or community-based CR, CR guidelines recommend delivery of the same established core CR components in these programs, including structured exercise, patient education and counseling. For example, in home-based CR, a patient would come in to the CR center for an initial assessment, during which patient safety for independent exercise would be established and an exercise prescription developed. Then, exercise training is performed without formal supervision, and regular contacts via phone or other technology (i.e., eCR) [8] are made to deliver the other components and review exercise. In community-based CR, local community exercise facilities are exploited, and CR staff go to these centers to deliver comprehensive services.

A series of Cochrane reviews have established the equivalent benefits of home and supervised CR [9], and there have also been reviews on the benefits of eCR (including greater patient adherence, suggesting they indeed mitigate patient barriers) [10,11,12]. There have been a few studies demonstrating the benefits of community-based CR as well [13,14,15]. While home-based CR may not be more cost-effective than supervised programs [9] (which indeed are very cost-effective and affordable) [16], eCR and community-based programs may well be. Moreover, recent data by our group demonstrate programs offering alternative models that treat a higher volume of patients [7], and this could be particularly true for eCR. Therefore, it is clear that delivery of alternative models should be optimized globally.

It is then surprising that there is little information in the literature regarding the proportion of programs offering these models, how they allocate patients to model (e.g., based on geography, risk of an adverse event) and the proportion treated in the various models, whether alternate models are reimbursed (among other potential barriers to delivery), and the degree of exploitation of new eCR possibilities, among other considerations. Studies in Canada suggest only approximately 10% of patients receive home-based CR, and unfortunately use was not associated with distance from centre, but was associated with disease severity [17,18]. Other studies around the world suggest that 15% of patients in Australia [19], 12% in New Zealand [20], 28% in Europe [21], and 38% in Mexico [22] participate in alternative models. Our recent literature review of national CR program surveys summarizes the proportion of programs delivering alternative models in countries where assessed [23].

Therefore, the objective of this study was to ascertain the models of CR delivered in countries around the globe (i.e., home [including eCR] and community-based), describe the nature of these models (e.g., dose, proportion of patients served, healthcare provider types delivering, basis for offering) and their barriers to delivery.

## 2. Experimental Section

This research was observational and cross-sectional in design. Protocol details are provided elsewhere [7]. Countries in the world where CR services were available were identified through reviews [24,25] as well as key informants, and identified leaders in these countries were sent an e-mail requesting their assistance administering the survey to each program therein.

Each program identified was emailed requesting their completion of the survey. Informed consent was secured through an online form. Data were collected confidentially through REDCap from June 2016 to July 2017. Contacts were sent two e-mail reminders, at 2-week intervals.

The study was reviewed by York University’s Office of Research Ethics (Toronto, Canada) and Mayo Clinic’s Institutional Review Board (Rochester, United States); both institutions provided an ethics approval exemption given the study methods. Informed consent was sought through an online consent form. Respondents were required to click a box to consent and proceed to complete the survey.

The sample consisted of all CR programs identified in the world, that offered services to patients following an acute cardiac event or hospitalization (i.e., Phase II). The inclusion criteria were CR programs that offered: (a) initial assessment, (b) structured exercise, and (c) at least one other strategy to control cardiovascular (CV) risk factors. All programs were contacted in countries known to have ≤250 CR programs. Where more existed (only the United States), a random sub-sample of 250 were contacted.

Development of the survey is described in detail elsewhere [26]. In short, items were based on previous national/regional CR program surveys [27,28,29]. Most items had forced-choice response options, and skip-logic was used to get more detail where applicable. This was pilot-tested in the Arab world and Canada [26], and is available elsewhere [30].

Specific survey items were the focus of this paper, namely regarding availability and the nature of alternative models. Alternative models comprised home-based (eCR where information and communications technology were used), community-based and hybrid programs. In regards to home-based programs, 13 questions were posed including a proportion of program patients served, forms of communication between patients and the program, and barriers. In regards to community-based programs, 12 questions similar to the home-based items were posed.

SPSS version 24 was used for analysis [31]. All initiated surveys were included. The number of responses for each question varied due to missing data (e.g., respondent did not answer a question due to lack of willingness or potential inapplicability, use of skip logic); for descriptive analyses, percentages were computed with the denominator being the number of programs offering a given model.

The proportion of countries offering each model type (i.e., by at least one program) was computed. To characterize model delivery by programs within countries, descriptive statistics were applied for all closed-ended items in the survey (e.g., frequencies with percentages, as well as means and standard deviations or medians with quartiles (Q25–Q75)). All open-ended responses were coded/categorized using an interpretive-descriptive approach by the first author and reviewed by the last [32]. Responses were then compared by country and World Health Organization (WHO) region; inferential comparisons were not undertaken due to small cell sizes.

## 3. Results

**Figure 1:**
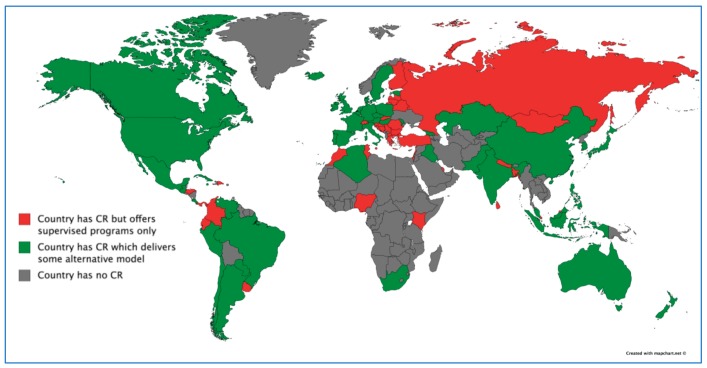
Countries That Offer Any Alternative Models of Cardiac Rehabilitation around the Globe. CR = cardiac rehabilitation.

There were 111/203 (54.7%) countries in the world with CR, of which data were collected in 93 (83.8%). Figure 1 displays the 92 countries without CR. Response rate by country is reported elsewhere [30].

Globally, 85 (76.6%) countries with CR (or 41.9% of all the countries in the world) offered supervised CR (*n* = 630 programs, 68.9%), and 51 (45.9%) countries with CR (or 25.1% of all the countries in the world) offered some alternative model (Figure 1; *n* = 285 programs, 31.1%). Twelve (1.1%) programs offered only alternative models (*n* = 8, 7.2% countries). This was significantly more common in high versus low and middle-income countries (see [33]). The proportion of responding programs offering home and community-based CR specifically is shown by country and WHO region in Supplemental Table S1.

Ninety-two (8.5%) programs (from 24 countries) reported delivering some form of hybrid model (where patients had some supervised CR and then transitioned to another setting). By WHO region this was: 1 (5.6%) in the African region, 25 (9.6%) in the Americas, 3 (12.5%) in the Eastern Mediterranean region, 43 (8.9%) in Europe, 1 (3.1%) in the South-East Asian region, and 19 (7.2%) in the Western Pacific region. Twenty-nine (4.3%) programs reported delivering some “other” form of CR, which included (*n* = 1 for each) rural satellites, shorter or longer programs, heart clubs/peer programs, and programs offered in primary care, among other responses. Whether alternative models were reimbursed by government or private healthcare insurance is displayed by country and WHO region in Supplemental Table S1. Finally, while maintenance program delivery was beyond the scope of the study, 256 (23.7%) respondents reported offering some form of patient follow-up post-program.

### 3.1. Supervised CR

Year first offered (1944), capacity of supervised CR programs (3373 programs in the world that can serve 1,675,270 patients/year) [7], healthcare provider types delivering (on average 5/program; most-commonly nurses, exercise specialists/physiotherapists), core components offered (most commonly exercise training and patient education) [30], are reported elsewhere, by country and WHO region.

With regard to the provision of resistance training (90.8% globally) is reported elsewhere [30]. Patient education was one of the most commonly offered components. Number of sessions provided to patients per complete program and duration of education sessions is shown by country and WHO region in Supplemental Table S2. Delivery of non-traditional forms of exercise (e.g., yoga, dance) and of women-only classes is also reported elsewhere [31].

### 3.2. Home-Based CR

Information about home-based CR in the 38 countries where it is offered is shown by WHO region in Table 1 and by country in Supplemental Table S1. Median dose globally was three sessions for a full program (Q25–Q75 = 1.0–4.0).

Patients were most often offered a home-based program on the basis of choice or transportation barriers/distance (Table 1), with “other” responses including research (*n* = 4; 2.4%), and mobility (*n* = 2, 1.2%). Materials provided to patients (i.e., education) are also shown, with “other” responses including psychosocial/ stress management materials, and remote cardiac monitoring (*n* = 1 for each). Provider types interacting with patients in home-based programs were most often exercise physiologists/physiotherapists or nurses (Table 2), with “other” provider types including dietitians (*n* = 7; 4.2%), kinesiologists (*n* = 5; 3.0%), occupational therapists (*n* = 4; 2.4%), social workers (*n* = 1; 0.6%), and pharmacists (*n* = 1; 0.6%).

**Figure 2:**
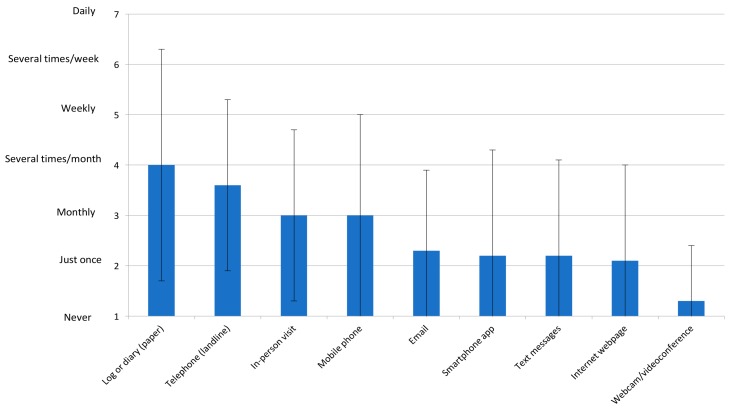
Forms of Communication in Home-Based Programs and Their Mean Frequency of Use Globally, *n* = 166.

Figure 2 displays the forms of communication with patients in home-based programs, along with frequency. This is shown by WHO region in Supplemental Figures S1–S6. No other forms of communication were reported. Barriers to using these communication tools are shown in Table 1, with one respondent also noting institutional policies restricted electronic communication with patients.

Overall, 106 (63.9%) responding programs reported using at least one form of information and communication technology (i.e., eCR) at least once per program with patients, and this was most commonly smartphone, email, and text messages (Figure 2). By WHO region, this was 2 (100.0%) for the Eastern Mediterranean Region, 34 (82.9%) for the Americas, 39 (62.9%) for Europe, 28 (51.9%) for the Western-Pacific, and 1 (50.0%) for Africa.

### 3.3. Community-Based CR

The nature of the community-based CR programs delivered in the 25 countries where it is offered is shown by WHO region in Table 2. Median dose globally was 20 sessions (Q25–Q75 = 9.6–36.0).

Providers responsible for supervising exercise sessions are also shown (most commonly exercise physiologist/physiotherapist). Where involved, physician type was most often cardiologists (*n* = 14, 1.3%), physiatrists (*n* = 6, 0.6%), and “other” provider types included dietitians (*n* = 6, 0.6%) and occupational therapists (*n* = 4, 0.4%). As also shown, programs most often offered patients a community-based program on the basis of choice, risk / indication, and distance/transportation.

### 3.4. Increasing Capacity of Alternative Models

**Figure 3:**
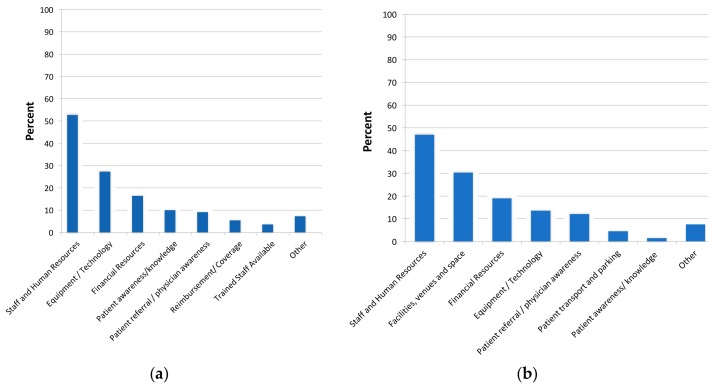
Needed elements to increase capacity of alternative model delivery. (**a**) home-based; (**b**) community-based.

As shown in Table 1, almost 40% of programs did not perceive they had sufficient capacity to meet demand in their home-based program, most commonly due to insufficient staff and funding. Respondents perceived they would need to increase their capacity to deliver home and community-based CR included staff, facilities/space, financial resources and equipment (Figure 3a,b respectively).

## 4. Discussion

Approximately half of countries in the world offer supervised CR, with alternative models of CR available in only one-quarter (or just less than half of those with any CR), despite the first such program starting 50 years ago. This is problematic given the grossly insufficient global CR capacity to meet the demand—capacity that could be drastically increased with broader provision of alternative models [7]. In the 15% of programs that even offered it, the median dose for home-based CR was a mere 3 sessions—a dose which is insufficient to achieve the reductions in morbidity and mortality associated with CR [35,36]. One-fifth of patients in CR are treated in unsupervised settings (but two-thirds in community-based, where available in the 10% of programs), yet there is no evidentiary basis on which to allocate patients to such models. So while appropriateness of this cannot be established, it is assumed that this is greatly constraining CR reach as only 5% of programs reported patient risk as the limiting factor.

The most-commonly used forms of communication in non-clinical CR settings were non-technological forms such as landlines, and paper logs/diaries, although in well over half of the programs patients came on site monthly. This is likely to ensure rapport and check clinical status for safety assurance. eCR has been introduced in about two-thirds of the programs. Similar to supervised programs [30], exercise physiologists/physiotherapists were commonly delivering services. Two-thirds of patients received patient education materials, and one-third a device to track physical activity. Use of telemetry was infrequent, which may be appropriate as there is no evidence it improves safety, and it comes with significant cost.

Given (a) it is expensive and often unnecessary to treat CVD patients in a hospital setting (and could expose patients to nosocomial infections), (b) that CR delivered in alternative setting is shown to be equivalent in benefit to supervised programs [9], and (c) that more patients can be treated in alternative settings, the clinical and policy implications of this first ever global survey on alternative CR delivery models are many. First, the capacity for alternative model delivery is very low, and this is likely limiting the reach of CR. The Western Pacific region appears to be the leader in delivering alternative models (followed by the Americas for home-based and Europe for community-based); we need to learn more about how these regions have achieved these heights, and apply their lessons in other jurisdictions. Where community-based services are offered, it appears that the programs are generally headquartered in the community and offer sufficient dose; more programs should be encouraged to establish community satellite centers. Barriers to greater provision were most often human resource-related. Funding and training (e.g., ICCPR Cardiovascular Rehabilitation Foundation Certifications [37]) for CR staff should be promoted.

Second, allocation to program model is currently haphazard, and again it would be useful for the global CR community to review available evidence and achieve consensus on a triage algorithm, taking into consideration the factors shown to be commonly-considered herein (i.e., patient preference, clinical considerations, logistical factors). For example, there was quite a bit of variation in terms of level of risk accepted to these models offered outside of clinical centers. It is assumed that most indicated patients (likely well more than the approximately 20% getting home-based) would be moderate or low-risk (i.e., only 5% of responding programs reported patient risk was a barrier to delivery of home-based CR; and a quarter of programs offering home-based could safely accept high-risk patients), and hence could safely receive CR services outside a clinical setting (including in the community—a setting that was not commonly exploited; this too warrants further investigation). The proportion of patients served in alternative models is not based on evidence but likely policy, given that a mere 12% of countries that offer CR reimburse alternative models. With an evidence-based allocation algorithm for alternative CR models established, programs could be encouraged to ascertain the proportion of indicated patients in their referral catchment appropriate for such models, and then work to ensure capacity meets this need. This would be facilitated by sharing of previously-developed and validated eCR models open source, successful advocacy for reimbursement of alternative models [38] (it is encouraging to note that there appears to be movement in this direction in the United States), as well as the development of ‘communities of practice’ to increase provider competence in delivering CR in non-traditional settings.

Third, while the survey failed to explicitly assess core components delivered in alternative models [31] (but only two-thirds of home-based patients received education materials), clearly the dose of three home-based CR sessions (although these were over 3 months, and this may not capture asynchronous communication between patients and program staff) is grossly insufficient. This would not ensure comprehensive delivery of all secondary prevention recommendations, to promote long-term patient adoption of a heart-healthy lifestyle and the achievement of all CVD risk factor targets [39], and hence the morbidity and mortality reductions associated with CR participation [1]. The global CR community should come together to agree on minimum standards for these models—standards which should then be tested empirically in various settings with regard to safety and efficacy.

Caution is necessary when interpreting the findings, particularly due to limits on generalizability. Firstly, response rates to online surveys are notoriously low. Second, it may not have been possible to identify all programs. For instance, smaller, community-based centers may have been missed if they did not have a website or engage with the CR community in their country. Thirdly and on a related note, programs affiliated with prominent academic centers may have been more readily identified for surveying. These centers may be more likely to offer alternative models than an average CR program, and therefore results may reflect somewhat greater provision of alternative models than reality.

Fourth, there are measurement issues. Items were self-reported, and a random subsample of programs were not audited to corroborate reporting. Moreover, many respondents did not complete all survey items, even in cases where they were applicable. This missing data may have introduced additional bias, such that results could reflect better delivery than reality if respondents did not respond if responses did not reflect guideline-based care. Finally, cell sizes were quite small regarding the nature of alternative models delivered. Therefore, proportions may not be reliable.

The limitations of alternative models of CR should also be considered. Some patients may prefer going to a hospital setting to exercise, for concerns over safety (which may or may not be valid) or for needed motivation [40]. With regard to the latter, for some patients, having less in-person contact with healthcare providers may result in less engagement in the program, and ultimately less heart-healthy behavior change. Some patients should also potentially only be treated in a supervised setting, not only for reasons of risk of an acute event during exercise, but for instance if they have clinical depression, communication barriers, or are at risk of falling during exercise.

## 5. Conclusions

In conclusion, through this first-ever global survey of CR programs, the fact that three-quarters of countries with CR offer supervised models, and almost one-half offer alternative models (38 countries offer home-based and 25 offer community) has been characterized for the first time around the world. Home-based models were least-frequently available in Europe and Western-Pacific regions, but where offered; 15% of programs in these countries offered the model, treating 21% of their total yearly patients. The scant number of home-based sessions were most commonly delivered by an exercise professional or physiotherapist, on a landline. The basis for offering alternative models was most-commonly patient choice, while model capacity was considered insufficient to meet patient need almost half the time. The CR community needs to develop evidence-based standards on which to allocate patients to alternative models, and then ensure sufficient capacity in such models, and that they are delivered in accordance with CR standards.

## Figures and Tables

**Table 1:** Characteristics of Home-Based Programs by World Health Organization Region.

*n* (%)/Mean ± Standard Deviation	African (*n* = 2)	Americas (*n* = 41)	EMR (*n* = 5)	Europe (*n* = 62)	SEAR (*n* = 2)	Western Pacific (*n* = 54)	Global (*n* = 166; 15.3% Total)
Year 1st program opened	2014	1979	2010	1986	2005	1980	1979
% pts served	20.0 ± 0.0 x̃ = 20.0 Q25–Q75 = 20.0–20.0	18.7 ± 18.3 x̃ = 10.0 Q25–Q75 = 5.0–34.8	12.5 ± 15.7 x̃ = 7.5 Q25–Q75 = 2.5–25.0	12.9 ± 11.8 x̃ = 10.0 Q25–Q75 = 5.0–25.0	55.0 ± 35.4 x̃ = 55.0 Q25–Q75 = 30.0–55.0	35.6 ± 30.8 x̃ = 22.5 Q25–Q75 = 10.0–60.0	21.4 ± 22.8 x̃ = 10.0 Q25–Q75 = 5.0–30.0
% perceive sufficient capacity ^§^	1 (50.0%)	19 (46.3%)	3 (60.0%)	36 (58.1%)	1 (50.0%)	28 (51.9%)	88 (53.0%)
*Not enough staff*	0 (0.0%)	11 (26.9%)	1 (20.0%)	15 (24.3%)	1 (50.0%)	14 (25.9%)	42 (25.3%)
*Not enough funding*	0 (0.0%)	7 (17.1%)	2 (40.0%)	13 (21.0%)	1 (50.0%)	8 (14.8%)	31 (18.7%)
*Pts too high-risk*	0 (0.0%)	2 (4.9%)	1 (20.0%)	0 (0.0%)	0 (0.0%)	3 (5.6%)	6 (5.2%)
Dose							
Number sessions pts prescribed per month ^a^	8.0 ± 0.0 x̃ = 8.0 Q25–Q75 = 8.0–8.0	3.2 ± 2.7 x̃ = 3.0 Q25–Q75 = 1.0–4.0	6.2 ± 4.6 x̃ = 5.0 Q25–Q75 = 2.0–11.0	4.9 ± 7.1 x̃ = 3.0 Q25–Q75 = 2.0–4.0	6.5 ± 7.8 x̃ = 6.5 Q25–Q75 = 1.0–6.5	3.9 ± 4.2 x̃ = 2.0 Q25–Q75 = 1.0–4.0	4.2 ± 5.3 x̃ = 3.0 Q25–Q75 = 1.0–4.0
Program duration (months)	4.0 ± 0.0 x̃ = 4.0 Q25–Q75 = 4.0–4.0	5.8 ± 3.9 x̃ = 4.0 Q25–Q75 = 3.0–6.5	2.5 ± 0.8 x̃ = 3.0 Q25–Q75 = 1.6–3.0	2.7 ± 2.0 x̃ = 2.0 Q25–Q75 = 2.0–3.0	-	2.3 ± 1.8 x̃ = 1.5 Q25–Q75 = 0.9–3.0	3.6 ± 3.1 x̃ = 3.0 Q25–Q75 = 2.0–4.0
Providers Interacting with Pts ^‖^							
Exercise physiologist or physiotherapist	2 (100.0%)	32 (72.0%)	0 (0.0%)	44 (72.1%)	2 (100.0%)	21 (38.9%)	101 (60.8%)
Nurse	0 (0.0%)	12 (29.3%)	3 (60.0%)	31 (50.0%)	0 (0.0%)	32 (59.3%)	78 (47.0%)
Physician	0 (0.0%)	10 (24.4%)	5 (100.0%)	14 (22.6%)	0 (0.0%)	14 (25.9%)	43 (25.9%)
Basis for Offering							
Patient choice	0 (0.0%)	36 (87.8%)	3 (60.0%)	45 (72.6%)	2 (100.0%)	35 (64.8%)	121 (72.9%)
Transportation barriers	1 (50.0%)	34 (82.9%)	5 (100.0%)	39 (62.9%)	2 (100.0%)	29 (53.7%)	110 (66.3%)
Distance	1 (50.0%)	35 (85.4%)	5 (100.0%)	33 (53.2%)	2 (100.0%)	32 (59.3%)	108 (65.1%)
Time or work constraints	0 (0.0%)	29 (70.7%)	2 (40.0%)	26 (41.9%)	2 (100.0%)	25 (46.3%)	84 (50.6%)
Risk stratification	0 (0.0%)	24 (58.5%)	1 (20.0%)	25 (40.3%)	1 (50.0%)	25 (46.3%)	76 (45.8%)
Patient indication	0 (0.0%)	20 (48.8%)	1 (20.0%)	22 (35.5%)	1 (50.0%)	23 (42.6%)	67 (40.4%)
Cost	1 (50.0%)	9 (22.0%)	3 (60.0%)	6 (9.7%)	2 (100.0%)	17 (31.5%)	38 (22.9%)
Exercise Monitoring							
Borg perceived exertion [34]	1 (50.0%)	20 (48.8%)	2 (40.0%)	28 (45.2%)	2 (100.0%)	17 (31.5%)	70 (42.2%)
Heart rate	1 (50.0%)	22 (53.7%)	1 (20.0%)	26 (41.9%)	1 (50.0%)	14 (25.9%)	65 (39.2%)
Telemetry	0 (0.0%)	0 (0.0%)	1 (20.0%)	6 (9.7%)	0 (0.0%)	9 (16.7%)	16 (9.6%)
Materials Provided							
Education materials (workbook, DVD, website)	1 (50.0%)	34 (82.9%)	3 (60.0%)	38 (61.3%)	1 (50.0%)	37 (68.5%)	114 (68.7%)
Activity tracker (accelerometer, pedometer)	0 (0.0%)	24 (58.5%)	1 (20.0%)	20 (32.3%)	1 (50.0%)	12 (22.2%)	58 (34.9%)
Resistance training materials (e.g., therabands)	1 (50.0%)	11 (26.8%)	1 (20.0%)	6 (9.7%)	1 (50.0%)	7 (13.0%)	27 (16.3%)
Level of Risk Accepted							
High	0 (0.0%)	12 (29.3%)	1 (20.0%)	18 (29.0%)	1 (50.0%)	8 (14.8%)	40 (24.1%)
Moderate	1 (50.0%)	27 (65.9%)	4 (80.0%)	42 (67.7%)	2 (100.0%)	23 (42.6%)	99 (59.6%)
Low	1 (50.0%)	36 (87.8%)	4 (80.0%)	48 (77.4%)	2 (100.0%)	30 (55.6%)	121 (72.9%)
Do not risk stratify	1 (50.0%)	2 (4.9%)	0 (0.0%)	1 (1.6%)	0 (0.0%)	10 (18.5%)	13 (7.8%)
Barriers to Communication with pts (% yes)							
Lack of pt access (e.g., no computer)	-	13 (31.7%)	5 (100.0%)	12 (19.4%)	1 (50.0%)	13 (24.1%)	44 (26.5%)
Logistical problems (e.g., internet connection)	-	16 (39.0%)	3 (60.0%)	7 (11.3%)	1 (50.0%)	11 (20.4%)	38 (22.9%)
Difficulty for staff	-	4 (9.8%)	2 (40.0%)	5 (8.7%)	0 (0.0%)	8 (14.8%)	19 (11.4%)

^a^ formal contact with cardiac rehabilitation staff. ^§^ Respondents responding ‘yes’ perceived their program to have sufficient capacity to meet need/demand in the home-base model reported in this row; respondents responding ‘no’ were asked to specify why they do not have sufficient capacity. These are shown in the subsequent three rows (italics). ^‖^ Total number of providers on staff reported elsewhere [30]; x̃ = median; - no response; Abbreviations: pts = patients; Acronyms: SEAR = South-East Asia region; EMR = Eastern Mediterranean region.

**Table 2:** Characteristics of Community-Based Programs by World Health Organization Region.

*n* (%)/Mean ± Standard Deviation	African (*n* = 1)	Americas (*n* = 21)	EMR (*n* = 1)	European (*n* = 54)	SEAR (*n* = 1)	Western Pacific (*n* = 31)	Global (*n* = 109; 10.1% of Total)
Year the 1st program opened	-	1982	2012	1979	-	1968	1968
% pts served	-	28.5 ± 27.0 x̃ = 20.0 Q25–Q75 = 5.0–42.5	10.0 ± 0.0 x̃ = 10.0 Q25–Q75 = 10.0–10.0	43.7 ± 30.6 x̃ = 40.0 Q25–Q75 = 20.0–69.0	50.0 ± 0.0 x̃ = 50.0 Q25–Q75 = 50.0–50.0	38.5 ± 38.6 x̃ = 20.0 Q25–Q75 = 5.0–83.8	38.1 ± 32.3 x̃ = 27.5 Q–25–75 = 10.0–61.3
Where provided							
Public centre	-	15 (71.4%)	1 (100.0%)	25 (46.3%)	0 (0.0%)	21 (67.7%)	62 (56.9%)
Private centre	-	2 (9.5%)	0 (0.0%)	17 (31.5%)	0 (0.0%)	4 (12.9%)	23 (21.1%)
Semi-private centre	-	0 (0.0%)	0 (0.0%)	6 (11.1%)	1 (100.0%)	1 (3.2%)	8 (7.3%)
Dose							
Sessions pts prescribed/month	-	9.1 ± 4.5 x̃ = 9.0 Q25–Q75 = 4.0–12.0	-	9.0 ± 9.1 x̃ = 8.0 Q25–Q75 = 4.0–10.0	2.0 ± 0.0 x̃ = 2.0 Q25–Q75 = 2.0–2.0	6.6 ± 5.3 x̃ = 5.0 Q25–Q75 = 2.0–11.0	8.3 ± 7.3 x̃ = 8.0 Q25–Q75 = 4.0–12.0
Duration (months)	-	5.5 ± 1.5 x̃ = 4.0 Q25–Q75 = 3.0–6.3	3.0 ± 0.0 x̃ = 3.0 Q25–Q75 = 3.0–3.0	2.9 ± 3.4 x̃ = 2.0 Q25–Q75 = 2.0–3.0	7.5 ± 0.0 x̃ = 7.5 Q25–Q75 = 7.5–7.5	3.4 ± 3.6 x̃ = 2.0 Q25–Q75 = 1.5–3.0	3.7 ± 3.7 x̃ = 2.5 Q25–Q75 = 1.0–3.0
Provider most responsible to supervise exercise sessions							
Exercise physiologist or physiotherapist	-	10 (47.6%)	1 (100.0%)	20 (37.0%)	0 (0.0%)	9 (29.0%)	40 (36.7%)
Nurse	-	3 (14.3%)	0 (0.0%)	16 (29.6%)	1 (100.0%)	8 (25.8%)	28 (25.7%)
Physician	-	2 (9.5%)	0 (0.0%)	6 (11.1%)	0 (0.0%)	5 (16.1%)	13 (11.9%)
Basis for Offering							
Patient choice	-	15 (71.4%)	0 (0.0%)	38 (70.4%)	1 (100.0%)	19 (61.3%)	73 (67.0%)
Risk stratification	-	13 (61.9%)	0 (0.0%)	26 (48.1%)	1 (100.0%)	11 (35.5%)	51 (46.8%)
Distance to main CR centre	-	16 (76.2%)	1 (100.0%)	18 (33.3%)	1 (100.0%)	13 (41.9%)	49 (45.0%)
Patient indication	-	8 (38.1%)	0 (0.0%)	24 (44.4%)	1 (100.0%)	13 (41.9%)	46 (42.2%)
Transportation barriers	-	14 (66.7%)	1 (100.0%)	17 (31.5%)	0 (0.0%)	11 (35.5%)	43 (39.4%)
Time or work constraints	-	15 (71.4%)	1 (100.0%)	13 (24.1%)	0 (0.0%)	11 (35.5%)	40 (36.7%)
Cost	-	3 (14.3%)	0 (0.0%)	6 (11.1%)	0 (0.0%)	2 (6.5%)	11 (10.1%)
Not having a main centre in a clinical setting	-	1 (4.8%)	0 (0.0%)	5 (9.6%)	0 (0.0%)	2 (6.5%)	8 (7.3%)
Capacity Indicators							
Number sessions offered per week	-	5.7 ± 8.1 x̃ = 3.0 Q25–Q75 = 1.8–6.3	-	4.7 ± 6.1 x̃ = 3.0 Q25–Q75 = 2.0–5.0	2.0 ± 0.0 x̃ = 2.0 Q25–Q75 = 2.0–0.0	3.8 ± 3.4 x̃ = 2.0 Q25–Q75 = 1.0–6.0	4.6 ± 6.0 x̃ = 3.0 Q25–Q75 = 2.0–6.0
Pts per session	-	16.9 ± 19.5 x̃ = 10.0 Q25–Q75 = 7.5–15.0	-	15.3 ± 14.8 x̃ = 12.0 Q25–Q75 = 9.3–15.0	10.0 ± 0.0 x̃ = 10.0 Q25–Q75 = 10.0–10.0	11.4 ± 8.1 x̃ = 10.0 Q25–Q75 = 8.0–15.0	14.6 ± 14.5 x̃ = 10.0 Q25–Q75 = 9.0–15.0
Exercise Monitoring							
Heart rate	-	10 (47.6%)	0 (0.0%)	23 (42.6%)	0 (0.0%)	14 (45.2%)	47 (43.1%)
Borg perceived exertion [34]	-	8 (38.1%)	1 (100.0%)	23 (42.6%)	0 (0.0%)	14 (45.2%)	46 (42.2%)
Telemetry	-	0 (0.0%)	0 (0.0%)	5 (9.6%)	0 (0.0%)	4 (12.9%)	9 (8.3%)
Level of Risk Accepted							
High	-	4 (19.0%)	0 (0.0%)	22 (40.7%)	0 (0.0%)	7 (22.6%)	33 (30.3%)
Moderate	-	13 (61.9%)	0 (0.0%)	37 (68.5%)	1 (100.0%)	18 (58.1%)	69 (63.3%)
Low	-	17 (81.0%)	1 (100.0%)	42 (77.8%)	1 (100.0%)	16 (51.6%)	77 (70.6%)
Do not risk stratify	-	2 (9.5%)	0 (0.0%)	2 (3.7%)	0 (0.0%)	5 (16.1%)	9 (8.3%)

x̃ = median; - no response; Abbreviations: pts = patients; Acronyms: SEAR = South-East Asia region; EMR = Eastern Mediterranean region.

## Supplementary Materials

The following are available online at http://www.mdpi.com/2077-0383/7/9/260/s1, Figure S1: Forms of Communication in Home-Based Programs and Their Frequency of Use in the African region, Figure S2: Forms of Communication in Home-Based Programs and Their Frequency of Use in the Americas, Figure S3: Forms of Communication in Home-Based Programs and Their Frequency of Use in the Eastern Mediterranean Region, Figure S4: Forms of Communication in Home-Based Programs and Their Frequency of Use in Europe, Figure S5: Forms of Communication in Home-Based Programs and Their Frequency of Use in the South-East Asia Region, Figure S6: Forms of Communication in Home-Based Programs and Their Frequency of Use in the Western Pacific region, Table S1: Delivery of alternative cardiac rehabilitation models by country, Table S2: Patient Education in Supervised Cardiac Rehabilitation by country.
