# Supplementary material for: Cardiac Rehabilitation Models around the Globe
Source: J Clin Med. 2018 Sep 7;7(9):260. doi: 10.3390/jcm7090260 (PMC6162832; doi:10.3390/jcm7090260)
Supplement: Supplementary file 1 [file jcm-07-00260-s001.pdf]

Figure S1: Forms of Communication in Home-Based Programs and Their Frequency of Use in the African region (N=1)

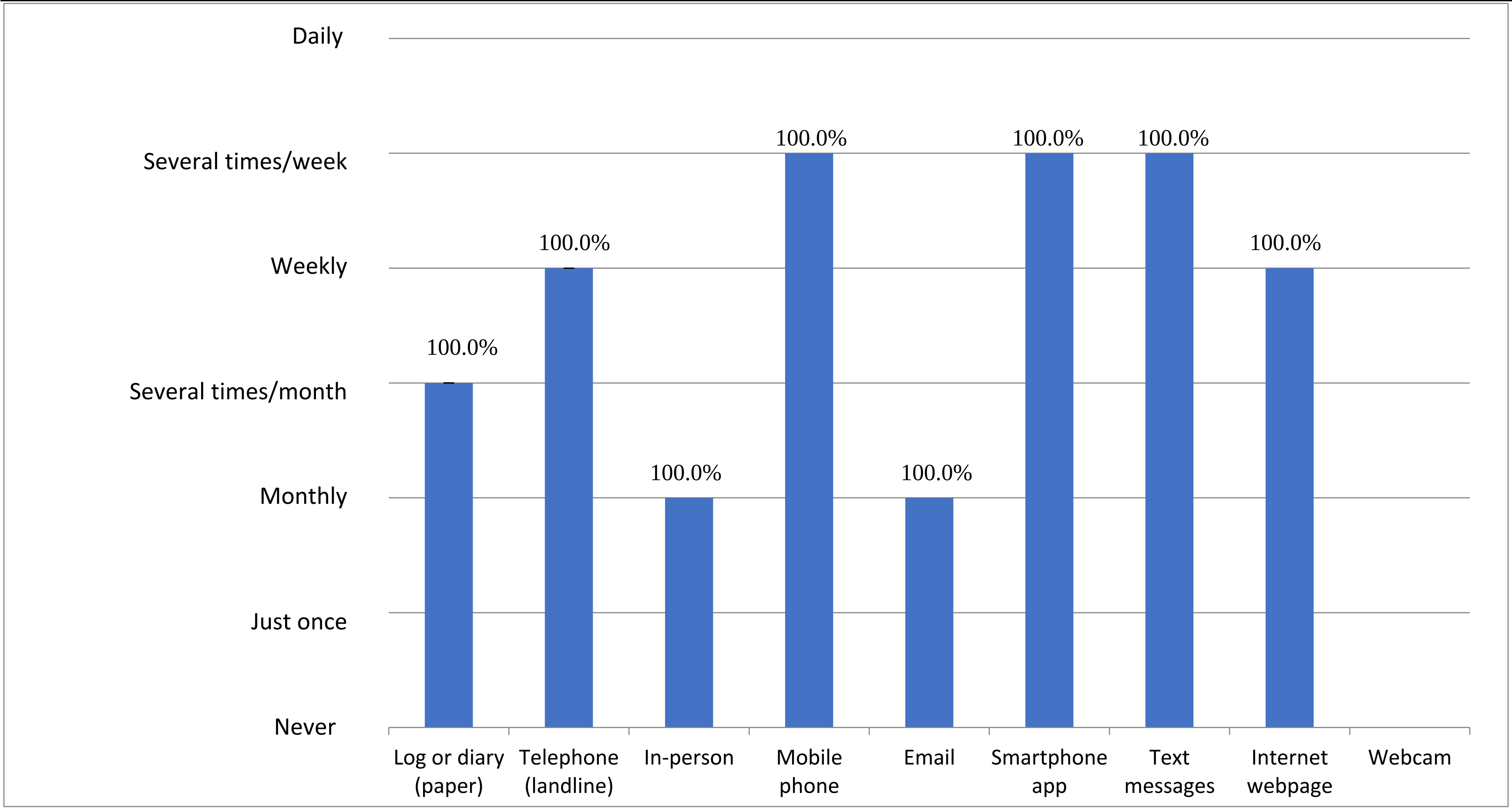

*no whiskers because only one respondent.
The percentages reflect the number of home-based programs that use this form of communication.

Figure S2 : Forms of Communication in Home-Based Programs and Their Frequency of Use in the Americas (N=37)

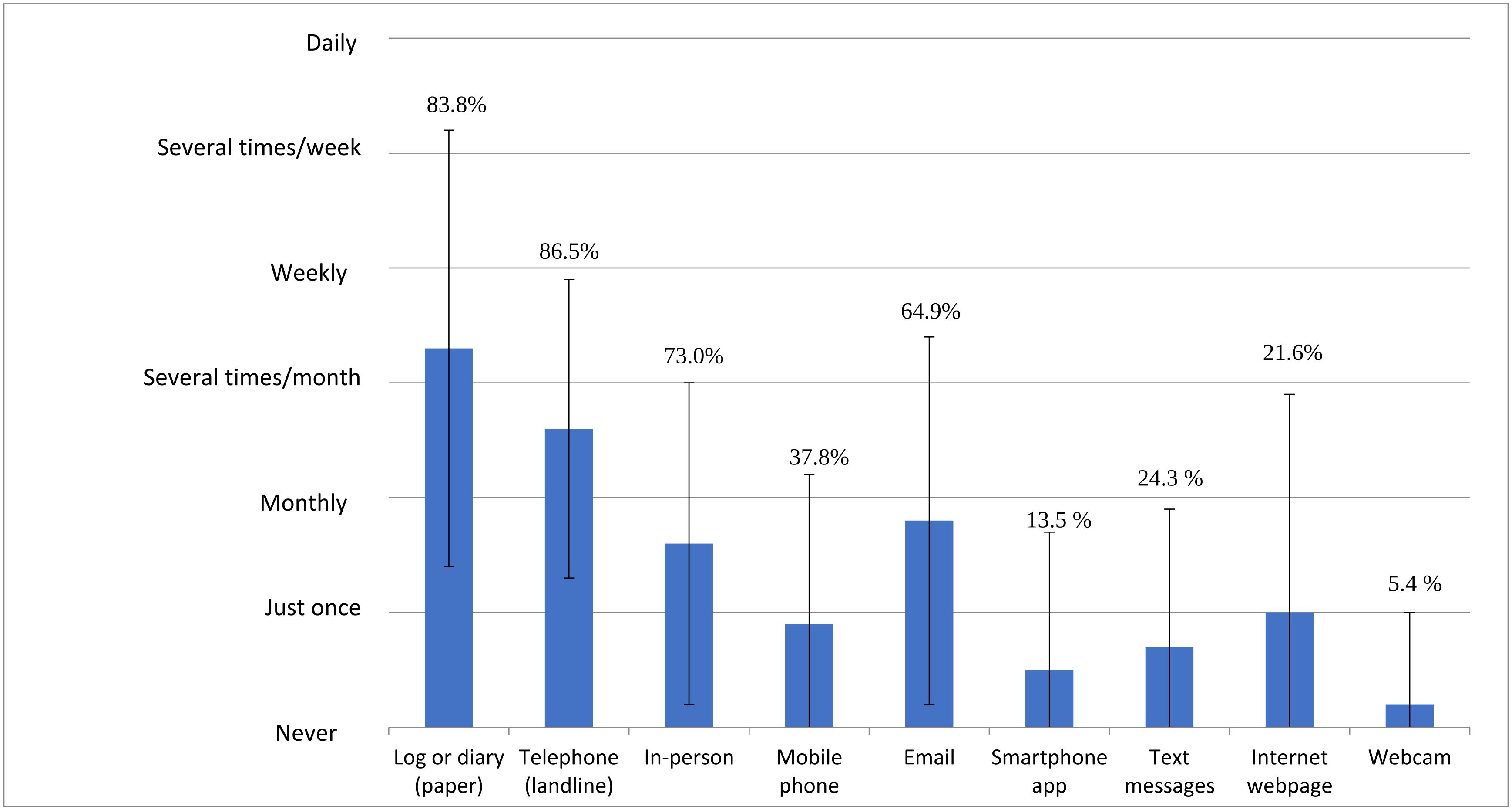

*whiskers represent standard deviations of each form of communication.
The percentages reflect the number of home-based programs that use this form of communication.

Figure S3: Forms of Communication in Home-Based Programs and Their Frequency of Use in the Eastern Mediterranean Region (N=4)

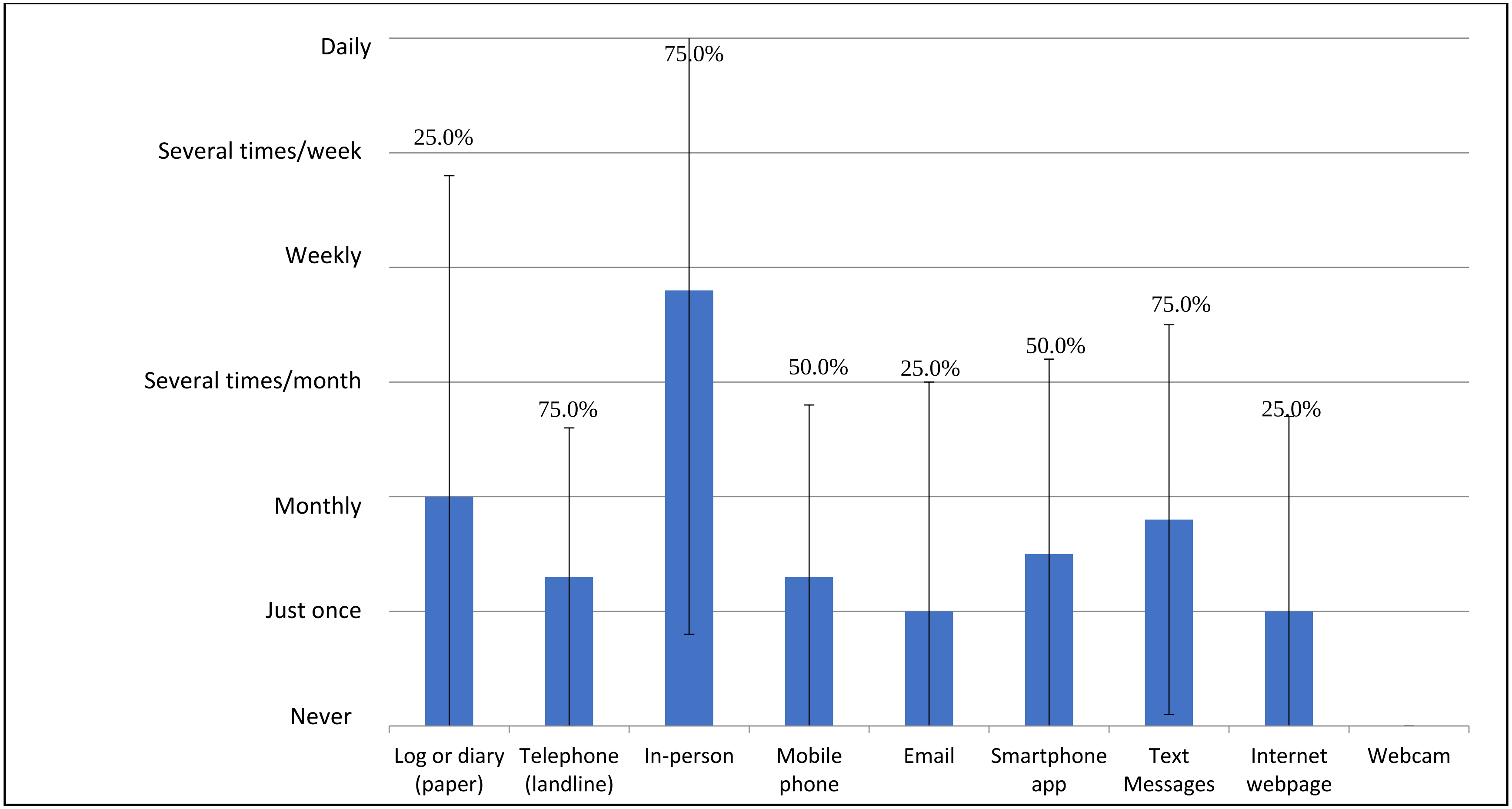

*whiskers represent standard deviations of each form of communication
The percentages reflect the number of home-based programs that use this form of communication.

Figure S4: Forms of Communication in Home-Based Programs and Their Frequency of Use in Europe (N=41)

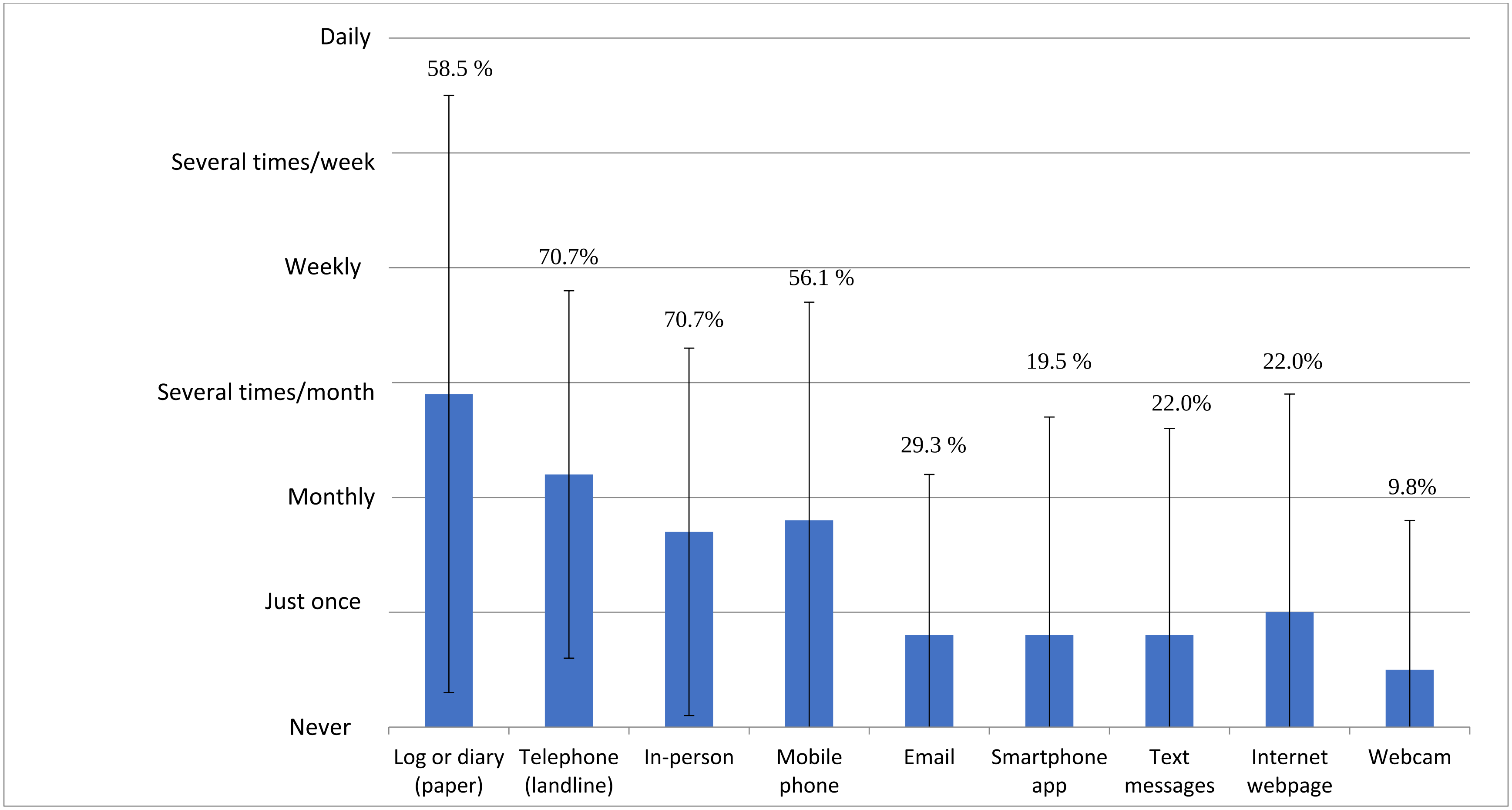

*whiskers represent standard deviations of each form of communication.
The percentages reflect the number of home-based programs that use this form of communication.

Figure S5: Forms of Communication in Home-Based Programs and Their Frequency of Use in the South-East Asia Region (N=2)

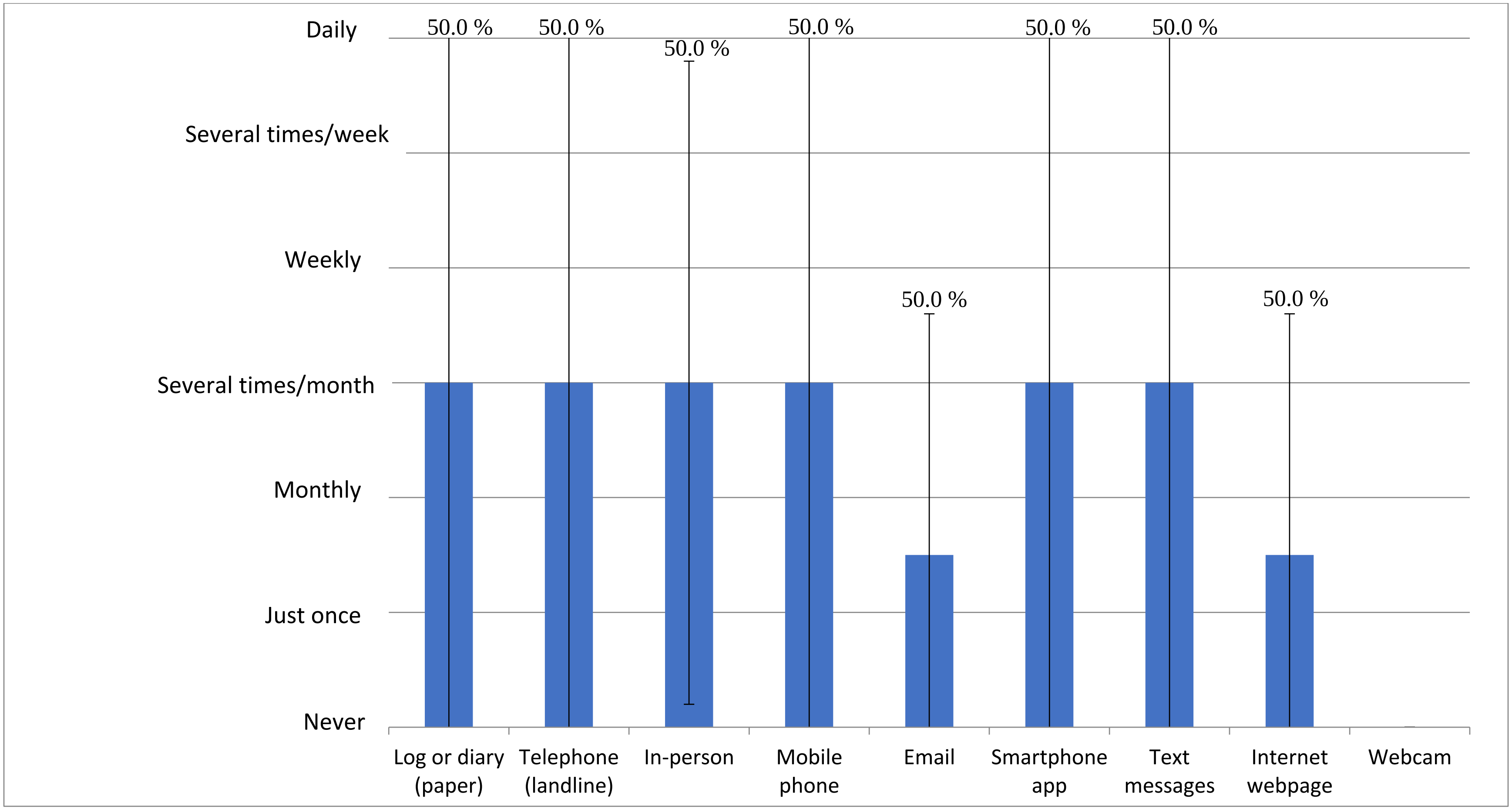

*whiskers represent standard deviations of each form of communication.
The percentages reflect the number of home-based programs that use this form of communication.

Figure S6: Forms of Communication in Home-Based Programs and Their Frequency of Use in the Western Pacific region (N=39)

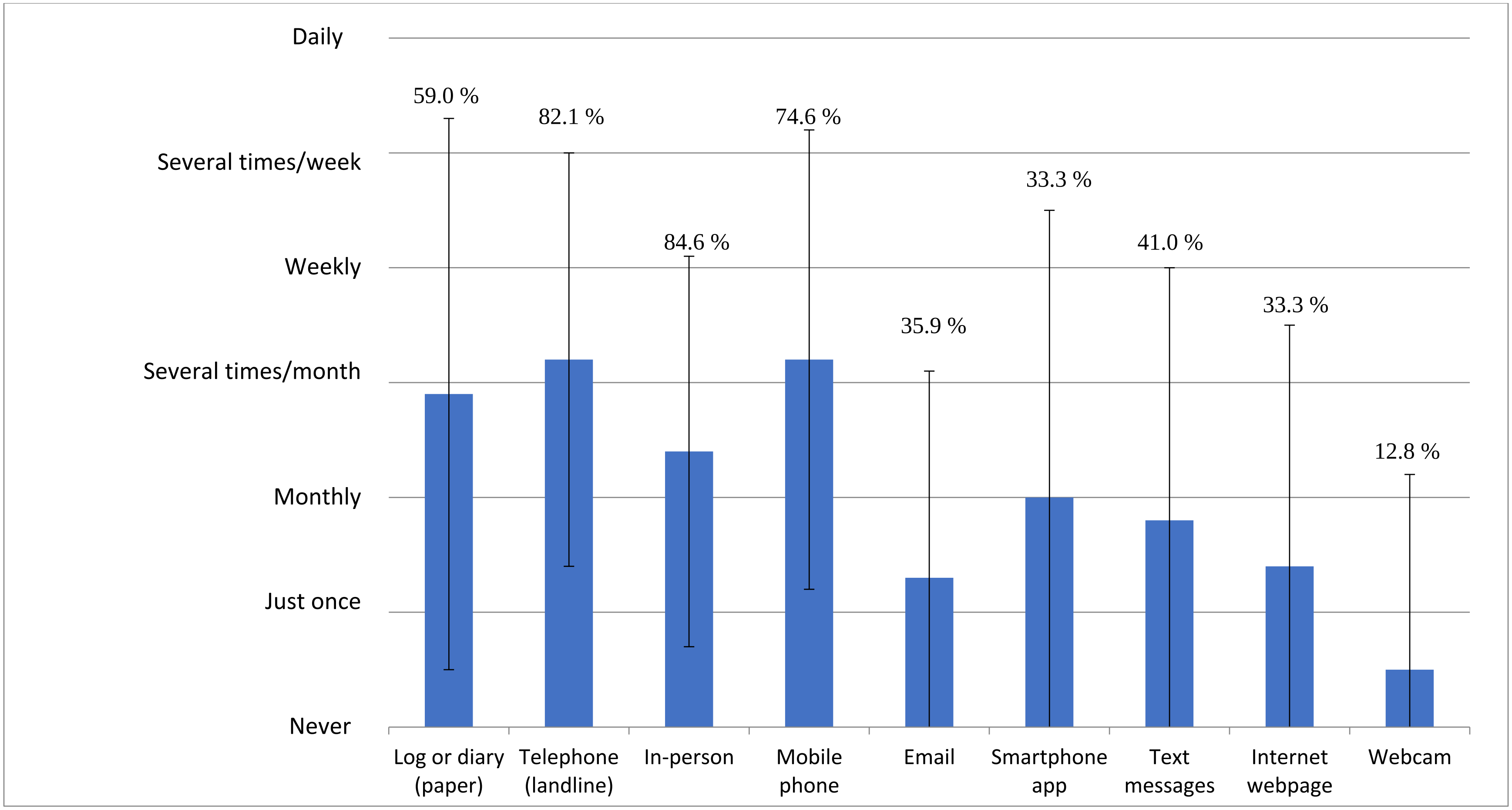

***whiskers represent standard deviations of each form of communication.**
The percentages reflect the number of home-based programs that use this form of communication.

Table S1: Delivery of alternative cardiac rehabilitation models by country

World Health Organization Region / Country	n	Offers Supervised CR	Offers Home-Based CR	Proportion Pts Served (%)	Home-based duration (wks)	Offers Community-Based CR	Alt. Models Reimbursed
**African**							
Algeria	1	-	1 (100.0%)	-	-	1 (100.0%)	-
Kenya	1	1 (100.0%)	-	-	-	-	0 (0.0%)
Mauritius	1	1 (100.0%)	-	-	-	-	0 (0.0%)
Nigeria	1	1 (100.0%)	-	-	-	-	0 (0.0%)
South Africa	14	13 (92.9%)	1 (7.1%)	20.0±0.0	16.0±0.0	0 (0.0%)	2 (14.3%)
Regional Average	*18*	*16 (88.9%)*	*2 (11.1%)*	*20.0±0.0*	*16.0±0.0*	*1 (5.6%)*	*2 (11.1%)*
**Americas**							
Argentina	3	3 (100.0%)	0 (0.0%)	-	-	0 (0.0%)	0 (0.0%)
Barbados	1	1 (100.0%)	-	-	-	-	0 (0.0%)
Bermuda	1	1 (100.0%)	1 (100.0%)	10.0±0.0	24.0±0.0	0 (0.0%)	1 (100.0%)
Brazil	30	23 (76.7%)	5 (16.7%)	23.3±23.1	38.0±13.1	1 (3.3%)	2 (6.7%)
Canada	57	46 (80.7%)	27 (47.4%)	14.8±17.8	21.8±16.5	13 (22.8%)	14 (24.6%)
Chile	1	1 (100.0%)	0 (0.0%)	-	-	0 (0.0%)	0 (0.0%)
Colombia	48	48 (100.0%)	-	-	-	-	0 (0.0%)
Costa Rica	6	6 (100.0%)	1 (16.7%)	30.0±0.0	12.0±0.0	2 (33.3%)	2 (33.3%)
Cuba	8	7 (87.5%)	2 (25.0%)	35.0±7.1	36.0±0.0	5 (83.3%)	6 (75.0%)
Curacao	1	1 (100.0%)	-	-	-	-	0 (0.0%)
Dominican Republic	1	1 (100.0%)	-	-	-	-	0 (0.0%)
Ecuador	2	2 (100.0%)	-	-	-	-	0 (0.0%)
Guatemala	2	2 (100.0%)	1 (50.0%)	-	12.0±0.0	0 (0.0%)	1 (50.0%)
Honduras	1	1 (100.0%)	-	-	-	-	0 (0.0%)

Jamaica	1	1 (100.0%)	0 (0.0%)	-	-	0 (0.0%)	1 (100.0%)
Mexico	9	9 (100.0%)	1 (11.1%)	50.0±0.0	8.0±0.0	0 (0.0%)	1 (11.1%)
Panama	1	1 (100.0%)	-	-	-	-	0 (0.0%)
Paraguay	3	3 (100.0%)	2 (66.7%)	40.0±0.0	36.0±0.0	0 (0.0%)	1 (33.3%)
Peru	7	7 (100.0%)	0 (0.0%)	-	-	0 (0.0%)	0 (0.0%)
United States of America	65	55 (84.6%)	0 (0.0%)	-	-	0 (0.0%)	1 (1.5%)
Uruguay	5	5 (100.0%)	-	-	-	-	0 (0.0%)
Venezuela	8	7 (87.5%)	1 (12.5%)	9.0±0.0	12.0±0.0	0 (0.0%)	0 (0.0%)
Regional Average	*261*	*231 (88.5%)*	*41 (15.7%)*	*18.7±18.3*	*23.2±15.7*	*21 (8.0%)*	*30 (11.5%)*
**Eastern Mediterranean**							
Afghanistan	1	0 (0.0%)	-	-	-	-	0 (0.0%)
Bahrain	1	1 (100.0%)	-	-	-	-	0 (0.0%)
Egypt	2	0 (0.0%)	-	-	-	-	0 (0.0%)
Iran	14	12 (85.7%)	5 (35.7%)	12.5±15.7	9.8±3.2	1 (7.1%)	3 (21.4%)
Lebanon	1	1 (100.0%)	-	-	-	-	0 (0.0%)
Morocco	1	0 (0.0%)	-	-	-	-	1 (100.0%)
Pakistan	2	2 (100.0%)	0 (0.0%)	-	-	0 (0.0%)	0 (0.0%)
Qatar	1	1 (100.0%)	-	-	-	-	0 (0.0%)
Tunisia	1	1 (100.0%)	-	-	-	-	0 (0.0%)
Regional Average	*24*	*18 (75.0%)*	*5 (20.8%)*	*12.5±15.7*	*9.8±3.2*	*1 (4.2%)*	*4 (16.7%)*
**Europe**							
Austria	5	5 (100.0%)	0 (0.0%)	-	-	1 (20.0%)	1 (20.0%)
Belarus	1	1 (100.0%)	-	-	-	-	0 (0.0%)
Belgium	9	7 (77.8%)	1 (11.1%)	2.0±0.0	12.0±0.0	2 (22.2%)	2 (22.2%)
Bosnia and Herzegovina	1	1 (100.0%)	-	-	-	-	0 (0.0%)
Bulgaria	1	1 (100.0%)	-	-	-	-	0 (0.0%)

Croatia	3	2 (66.7%)	-	-	-	-	1 (33.3%)
Czech Republic	6	4 (66.6%)	3 (60.0%)	-	11.0±1.4	0 (0.0%)	0 (0.0%)
Denmark	8	6 (75.0%)	1 (12.5%)	10.0±0.0	12.0±0.0	0 (0.0%)	3 (60.0%)
England	57	43 (75.4%)	17 (29.8%)	7.7±10.1	12.4±14.7	17 (29.8%)	13 (22.8%)
Estonia	2	1 (50.0%)	2 (100.0%)	5.0±0.0	12.0±0.0	0 (0.0%)	0 (0.0%)
Finland	11	3 (27.3%)	-	-	-	-	3 (27.3%)
France	16	11 (68.8%)	0 (0.0%)	-	-	1 (6.3%)	0 (0.0%)
Georgia	13	6 (46.2%)	1 (7.7%)	25.0±0.0	10.0±0.0	1 (7.7%)	1 (7.7%)
Germany	34	25 (73.5%)	0 (0.0%)	-	-	1 (2.9%)	6 (17.6%)
Greece	4	4 (100.0%)	-	-	-	-	0 (0.0%)
Hungary	20	15 (75.0%)	1 (5.0%)	10.0±0.0	6.0±0.0	2 (10.0%)	1 (5.0%)
Iceland	4	3 (75.0%)	0 (0.0%)	-	-	0 (0.0%)	1 (25.0%)
Ireland	7	5 (71.4%)	2 (28.6%)	-	-	1 (14.3%)	2 (28.6%)
Israel	6	5 (83.3%)	-	-	-	-	0 (0.0%)
Italy	70	51 (72.9%)	3 (4.3%)	18.3±12.6	6.0±0.0	6 (8.6%)	6 (8.6%)
Kazakhstan	1	-	1 (100.0%)	-	-	1 (100.0%)	-
Latvia	1	0 (0.0%)	-	-	-	-	0 (0.0%)
Lithuania	9	4 (44.4%)	-	-	-	-	0 (0.0%)
Macedonia	1	0 (0.0%)	-	-	-	-	0 (0.0%)
Malta	1	1 (100.0%)	-	-	-	-	0 (0.0%)
Netherlands	29	11 (37.9%)	2 (6.9%)	15.0±14.1	26.0±0.0	1 (3.4%)	3 (10.3%)
Northern Ireland	10	10 (100.0%)	4 (40.0%)	-	-	0 (0.0%)	0 (0.0%)
Poland	21	10 (47.6%)	0 (0.0%)	-	-	0 (0.0%)	2 (9.5%)
Portugal	21	17 (81.0%)	1 (4.8%)	1.0±0.0	-	0 (0.0%)	0 (0.0%)
Republic of Moldova	1	0 (0.0%)	1 (100.0%)	10.0±0.0	12.0±0.0	0 (0.0%)	0 (0.0%)
Romania	2	2 (100.0%)	-	-	-	-	0 (0.0%)

Russian Federation	3	1 (33.3%)	-	-	-	-	1 (33.3%)
Scotland	24	19 (79.2%)	12 (50.0%)	17.1±9.9	9.2±2.3	8 (33.3%)	1 (4.2%)
Serbia	2	1 (50.0%)	-	-	-	-	0 (0.0%)
Slovak Republic	1	1 (100.0%)	-	-	-	-	0 (0.0%)
Slovenia	2	2 (100.0%)	-	-	-	-	0 (0.0%)
Spain	47	41 (87.2%)	6 (12.8%)	24.0±17.8	8.0±0.0	4 (8.5%)	8 (17.0%)
Sweden	1	1 (100.0%)	0 (0.0%)	-	-	0 (0.0%)	0 (0.0%)
Switzerland	4	2 (50.0%)	-	-	-	-	0 (0.0%)
Turkey	9	7 (77.8%)	-	-	-	-	0 (0.0%)
Wales	16	14 (87.5%)	4 (25.0%)	5.5±6.4	12.0±5.7	8 (50.0%)	1 (6.3%)
Regional Average	*484*	*343 (70.9%)*	*62 (12.8%)*	*12.9±11.8*	*10.8±7.9*	*54 (11.2%)*	*56 (11.6%)*
**South-East Asia**							
Bangladesh	1	1 (100.0%)	0 (0.0%)	-	-	0 (0.0%)	0 (0.0%)
India	18	13 (72.2%)	2 (11.1%)	55.0±35.4	-	0 (0.0%)	1 (5.6%)
Indonesia	10	9 (90.0%)	0 (0.0%)	-	-	1 (10.0%)	0 (0.0%)
Nepal	1	1 (100.0%)	-	-	-	-	0 (0.0%)
Sri Lanka	2	2 (100.0%)	-	-	-	-	0 (0.0%)
Regional Average	*32*	*26 (81.3%)*	*2 (6.3%)*	*55.0±35.4*	-	*1 (3.1%)*	*1 (3.1%)*
**Western-Pacific**							
Australia	85	71 (83.5%)	22 (25.9%)	16.7±13.8	8.4±9.2	10 (11.8%)	19 (22.3%)
Brunei Darussalam	2	2 (100.0%)	-	-	-	-	0 (0.0%)
China	83	70 (84.3%)	14 (16.9%)	57.7±30.1	7.9±7.2	6 (7.2%)	4 (4.8%)
Japan	9	6 (66.7%)	1 (11.1%)	10.0±0.0	4.0±0.0	3 (33.3%)	3(33.3%)
Malaysia	4	3 (75.0%)	2 (50.0%)	20.0±0.0	8.0±5.7	1 (25.0%)	0 (0.0%)
Mongolia	1	1 (100.0%)	-	-	-	-	-

New Zealand	27	22 (81.5%)	7 (25.9%)	10.0±0.0	10.0±12.1	11 (40.7%)	6 (22.2%)
Philippines	10	10 (100.0%)	1 (10.0%)	15.0±0.0	-	0 (0.0%)	0 (0.0%)
Singapore	7	7 (100.0%)	-	-	-	-	1 (14.3%)
South Korea	12	12 (100.0%)	5 (41.7%)	60.0±35.2	10.0±4.0	0 (0.0%)	4 (25.0%)
Taiwan	23	17 (73.9%)	2 (8.7%)	27.5±31.8	16.0±5.7	0 (0.0%)	0 (0.0%)
Regional Average	*263*	*221 (84.0%)*	*54 (20.5%)*	*35.6±30.8*	*9.1±7.2*	*31 (11.8%)*	*37 (14.1%)*
**Global Average**	**1082**	**855 (79.0%)**	**166 (15.3%)**	**21.4±22.8**	**14.5±12.4**	**109 (10.1%)**	**130 (12.0%)**

- no response
Abbreviations: pts=patients; wks=weeks; Alt=alternative.
Acronyms: CR=cardiac rehabilitation

Table S2: Patient Education in Supervised Cardiac Rehabilitation by country

**World Health Organization Region**<br>Country<br>Mean ± std. deviation	**n**	**Number education sessions offered to patients per program**	**Education session duration (min.)**	**Total Education Dose (min.)**
**African**				
Algeria	1	-	-	
Kenya	1	2.0±0.0	20.0±0.0	40.0
Mauritius	1	-	35.0±0.0	-
Nigeria	1	12.0±0.0	45.0±0.0	540.0
South Africa	14	13.0±12.0	30.5±16.9	396.5
Regional Mean	*18*	*12.1±11.3*	*31.1±15.7*	*376.3*
**Americas**				
Argentina	3	3.0±0.0	52.5±10.6	157.6
Barbados	1	2.0±0.0	15.0±0.0	30.0
Bermuda	1	7.0±0.0	120.0±0.0	840.0
Brazil	30	6.0±10.5	45.1±29.6	270.6
Canada	57	11.6±11.6	62.6±30.8	
Chile	1	4.0±0.0	60.0±0.0	240.0
Colombia	48	6.0±6.7	49.1±31.3	294.6
Costa Rica	6	11.7±7.1	35.0±18.2	409.5
Cuba	8	12.0±10.2	37.5±17.3	450.0
Curacao	1	-	30.0±0.0	-
Dominican Republic	1	12.0±0.0	30.0±0.0	360.0
Ecuador	2	10.0±7.1	34.0±15.6	340.0
Guatemala	2	11.0±1.4	40.0±28.3	440.0
Honduras	1	2.0±0.0	30.0±0.0	60.0
Jamaica	1	-	-	-
Mexico	9	8.1±8.1	34.4±20.5	278.6
Panama	1	-	0.0±0.0	-
Paraguay	3	5.0±0.0	15.0±5.0	75.0
Peru	7	12.8±6.2	75.4±34.9	965.1
United States of America	65	14.6±12.2	42.1±29.4	614.7
Uruguay	5	3.7±0.6	44.0±31.3	162.8
Venezuela	8	10.4±3.4	46.9±12.5	487.8
Regional Mean	*261*	*9.9±11.1*	*48.4±30.2*	*479.2*
**Eastern Mediterranean**				

Afghanistan	1	-	120.0±0.0	-
Bahrain	1	11.0±0.0	30.0±0.0	330.0
Egypt	2	1.0±0.0	15.0±0.0	15.0
Iran	14	6.0±5.5	40.4±20.9	240.0
Lebanon	1	20.0±0.0	90.0±0.0	1800.0
Morocco	1	20.0±0.0	45.0±0.0	900.0
Pakistan	2	4.5±0.7	70.0±70.7	315.0
Qatar	1	3.0±0.0	30.0±0.0	90.0
Tunisia	1	5.0±0.0	20.0±0.0	100.0
Regional Mean	*24*	*7.1±6.4*	*46.4±32.3*	*329.4*
**Europe**				
Austria	5	36.8±62.2	68.8±44.2	2531.8
Belarus	1	-	-	-
Belgium	9	10.7±15.3	98.6±28.5	1055.0
Bosnia and Herzegovina	1	1.0±0.0	45.0±0.0	45.0
Bulgaria	1	2.0±0.0	10.0±0.0	20.0
Croatia	3	12.3±4.6	43.3±2.9	532.6
Czech Republic	6	4.2±4.6	22.0±22.6	92.4
Denmark	8	6.4±4.0	111.0±77.7	710.4
England	57	7.6±5.0	56.4±50.1	428.6
Estonia	2	2.0±0.0	30.0±0.0	60.0
Finland	11	18.0±18.4	82.5±21.2	1485.0
France	16	9.8±5.4	55.4±16.3	542.9
Georgia	13	8.4±6.8	20.0±8.4	168.0
Germany	34	20.6±30.7	54.5±41.0	1122.7
Greece	4	5.0±4.1	13.8±12.5	69.0
Hungary	20	8.3±6.1	36.3±13.1	301.3
Iceland	4	7.3±3.7	51.3±16.0	374.5
Ireland	7	12.6±3.0	58.5±3.4	737.1
Israel	6	1.8±0.8	54.0±25.1	97.2
Italy	70	6.9±7.4	48.5±26.1	334.7
Kazakhstan	1	-	-	-
Latvia	1	3.0±0.0	30.0±0.0	90.0
Lithuania	9	6.3±5.5	49.2±50.2	94.5
Macedonia	1	2.5±0.0	15.0±0.0	37.5
Malta	1	5.0±0.0	60.0±0.0	300.0

Netherlands	29	5.6±3.2	69.2±36.1	387.5
Northern Ireland	10	8.1±2.3	43.0±8.6	348.3
Poland	21	5.0±4.1	36.0±11.3	180.0
Portugal	21	13.7±11.5	56.1±21.2	768.6
Republic of Moldova	1	10.0±0.0	30.0±0.0	300.0
Romania	2	10.0±0.0	27.5±3.5	275.0
Russian Federation	3	0.0±0.0	0.0±0.0	-
Scotland	24	6.3±3.3	42.0±17.3	894.6
Serbia	2	5.5±3.5	60.0±0.0	330.0
Slovak Republic	1	2.0±0.0	45.0±0.0	90.0
Slovenia	2	56.0±62.2	37.5±31.8	2100.0
Spain	47	9.5±7.2	51.2±15.3	486.4
Sweden	1	-	60.0±0.0	-
Switzerland	4	12.0±0.0	45.0±0.0	540.0
Turkey	9	16.3±12.9	33.8±20.3	550.9
Wales	16	7.6±4.3	53.6±24.1	407.4
Regional Mean	*484*	*9.6±13.5*	*51.2±32.4*	*491.5*
**South-East Asia**				
Bangladesh	1	5.0±0.0	20.0±0.0	100.0
India	18	3.9±3.0	24.3±11.3	94.7
Indonesia	10	1.9±1.8	24.1±18.0	45.8
Nepal	1	6.0±0.0	60.0±0.0	360.0
Sri Lanka	2	8.0±0.0	45.0±21.2	360.0
Regional Mean	*32*	*3.5±2.9*	*25.8±13.6*	*90.3*
**Western Pacific**				
Australia	85	7.8±3.6	54.2±15.0	422.8
Brunei Darussalam	2	14.0±2.8	67.5±10.6	945.0
China	83	4.2±5.4	31.7±21.1	133.1
Japan	9	5.2±7.3	38.6±16.8	200.7
Malaysia	4	5.3±3.1	35.0±8.7	185.5
Mongolia	1	-	-	-
New Zealand	27	5.8±2.4	72.3±29.9	419.3
Philippines	10	5.4±3.7	29.0±13.7	156.6
Singapore	7	4.4±4.0	38.6±24.3	169.8

South Korea	12	3.0±3.0	23.3±6.2	69.,9
Taiwan	23	7.6±12.0	22.9±16.3	174.0
Regional Mean	*263*	*5.0±5.6*	*42.4±24.4*	*212.0*
**Global Mean**	**1082**	**8.5±10.8**	**47.0±29.7**	**399.5**

- no response
Abbreviations: std=standard; min=minimum.
